# Syndromic surveillance: two decades experience of sustainable systems – its people not just data!

**DOI:** 10.1017/S0950268819000074

**Published:** 2019-02-22

**Authors:** Gillian E. Smith, Alex J. Elliot, Iain Lake, Obaghe Edeghere, Roger Morbey, Mike Catchpole, David L. Heymann, Jeremy Hawker, Sue Ibbotson, Brian McCloskey, Richard Pebody, Public Health England Real-time Syndromic Surveillance Team

**Affiliations:** 1 Real-time Syndromic Surveillance Team, Field Service, National Infection Service, Public Health England, Birmingham, UK; 2 Health Protection Research Unit in Emergency Preparedness and Response, National Institute for Health Research, London, UK; 3 School of Environmental Sciences, University of East Anglia, Norwich, UK; 4 Field Epidemiology West Midlands, Field Service, National Infection Service, Public Health England, Birmingham, UK; 5 European Centre for Disease Control, Stockholm, Sweden; 6 London School of Hygiene and Tropical Medicine, London, UK; 7 West Midlands Centre, Public Health England, Birmingham, UK; 8 Global Health, Public Health England, London, UK; 9 Respiratory Diseases Department, National Infection Service, Public Health England, London, UK

**Keywords:** Public health, real-time, syndromic surveillance

## Abstract

Syndromic surveillance is a form of surveillance that generates information for public health action by collecting, analysing and interpreting routine health-related data on symptoms and clinical signs reported by patients and clinicians rather than being based on microbiologically or clinically confirmed cases. In England, a suite of national real-time syndromic surveillance systems (SSS) have been developed over the last 20 years, utilising data from a variety of health care settings (a telehealth triage system, general practice and emergency departments). The real-time systems in England have been used for early detection (e.g. seasonal influenza), for situational awareness (e.g. describing the size and demographics of the impact of a heatwave) and for reassurance of lack of impact on population health of mass gatherings (e.g. the London 2012 Olympic and Paralympic Games).We highlight the lessons learnt from running SSS, for nearly two decades, and propose questions and issues still to be addressed. We feel that syndromic surveillance is an example of the use of ‘big data’, but contend that the focus for sustainable and useful systems should be on the added value of such systems and the importance of people working together to maximise the value for the public health of syndromic surveillance services.

## Introduction: the practical value of syndromic surveillance

Syndromic surveillance is a form of surveillance that generates information for public health action by collecting, analysing and interpreting routine health-related data. Most commonly these data are symptoms and clinical signs reported by patients and clinicians rather than being based on microbiologically or clinically confirmed cases [1].When syndromic surveillance systems (SSS) were first established in the mid-1990s [2, 3], there was a particular emphasis on the monitoring of influenza activity and a further impetus because of the potential utility for the early detection of bioterrorist events in the wake of the terrorist attack in the USA in September 2001 [4].

In England, a suite of national real-time SSS have been developed incrementally since 1998, utilising data from a variety of health care settings (a telehealth triage system, general practice and emergency departments). These systems use descriptive methods and bespoke statistical modelling [5, 6] to detect a higher consultation/call activity than expected compared with historical trend data by syndrome and geography. If such increases are assessed to have potential public health implications, then the syndromic surveillance team alert public health colleagues in order to take public health action (Box 1) [7].

Box 1: Overview of the Public Health England syndromic surveillance service

**Figure:**
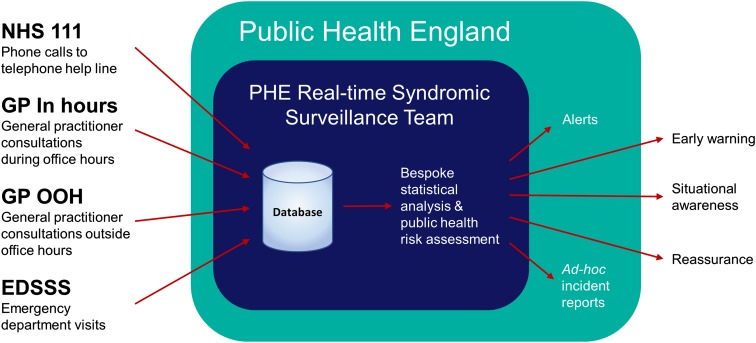

In England, key influenza-related indicators have been developed and SSS are used each winter for influenza surveillance and, alongside other sources of health intelligence including microbiological testing, play an important role in describing the onset of activity, spread and impact of influenza on the population each season. The SSS provide particular value in describing the impact of influenza on a variety of health care settings and were widely used to detect and monitor the H1N1(pdm09) pandemic (Box 2) [8]. Over the years the systems have been used to monitor the impact of a wide range of infectious and non-infectious hazards including seasonal norovirus activity, heatwaves, extreme cold weather, mass gatherings, air pollution events (Box 2) [9–12]. During the London 2012 Olympic and Paralympic Games, the SSS were used daily alongside enhanced microbiological and event-based surveillance to detect public health incidents, and also to particularly provide reassurance about lack of outbreaks across London and the rest England (Box 2) [13, 14]. Over recent years, the English SSS have also been used to monitor the impact on the population of the introduction of new vaccine programmes, e.g. rotavirus vaccine for infants in England and the subsequent impact on reducing GP consultations for gastroenteritis [15].

Box 2: Selected examples of the public health use of English syndromic surveillance systems

Weekly GP consultation rate of influenza-like illness during the early and peak phase of the H1N1(pdm09) pandemic across England.

**Figure:**
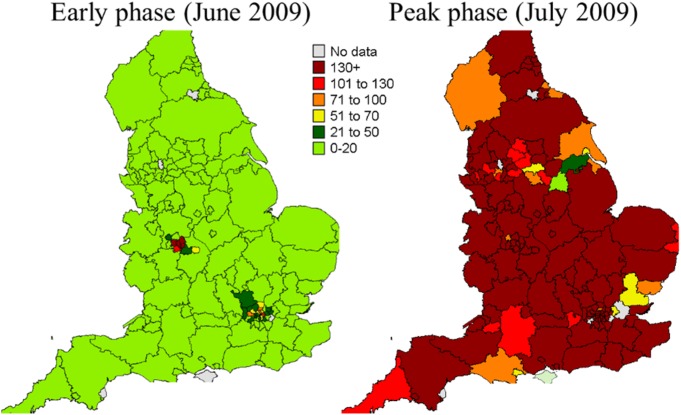

Extract from daily syndromic surveillance Olympic report illustrating reassurance of no public health threats.

**Figure:**
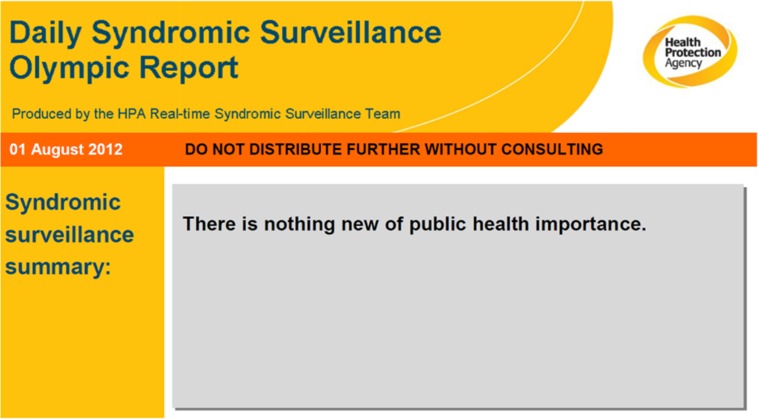

Increases in emergency department attendances for asthma/wheeze/difficulty breathing symptoms during two air pollution episodes, 2014.

**Figure:**
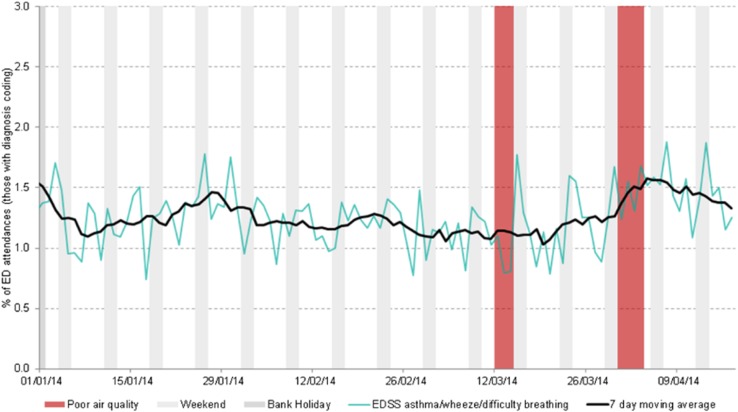

In summary, the real-time systems in England have been used for early detection (e.g. seasonal influenza and to support response planning), for situational awareness (e.g. describing the size and demographics of the impact of a heatwave) and for reassurance of lack of impact on population health of mass gatherings (e.g. the 2012 Olympic and Paralympic Games).

There has been considerable emphasis in the literature on the statistical methodologies and algorithms used to underpin SSS, with much of this work using synthetic and modelled data [16–20]. In addition, there are a number of publications on the detection of discrete events, e.g. cryptosporidiosis outbreaks. However, little has been published on the practical experience of establishing and operating such systems, and on how the outputs of SSS can be integrated into wider public health action and response.

In this review, we aim to highlight the lessons learnt from running SSS, for nearly two decades, and propose questions and issues still to be addressed. The authors include those who have commissioned SSS; led the design, implementation and maintenance of the systems; and the ‘end users’ of the outputs including national incident directors, and the opinions are a synthesis of their experience and views. We focus on lessons learnt from the English systems, but refer to SSS in other countries where relevant.

## Lessons learnt for syndromic surveillance

Whilst developing and using SSS, we have learnt a number of lessons which may be of value for those planning for, establishing and maintaining such systems. We have divided these lessons into those relating to the purpose and aims of SSS, relationships with colleagues and infrastructure of SSS.

### Lessons: purpose and aims

In lessons relating to purpose and aims, we highlight the importance of: integration of SSS within a public health system, public health colleagues driving the systems and SSS being multi-purpose.

Although there has been much emphasis in the literature on analytical methodologies and algorithms [16–20], which are important for ensuring the routine operation of SSS, it is essential that the primary focus should remain on the public health utility of these systems. Surveillance has been defined as providing ‘information for action’ and so it is important that the information produced by SSS is integrated within a public health system, which is able to take appropriate public health action as a result of the information generated, e.g. in Public Health England, the Real-time Syndromic Surveillance Team (ReSST) is part of the organisation which is able to take national and local public health action if an outbreak/incident is detected. In this way, SSS do not ‘stand-alone’ as ‘databases’, collecting data and generating statistical alarms, but rather are an integral part of a managed public health system. The connection between SSS operators and their public health stakeholders is key to ensuring that public health action needs to drive the scope and outputs of SSS. The SSS complement and augment a variety of traditional surveillance systems in order to provide wider intelligence about a public health issue or incident, e.g. early warning, or a more granular picture geographically or by age.

Sustainable systems (those which operate over a sustained period of time, are flexible and not established solely for a discrete event) focus on the needs of public health personnel who may make use of the syndromic surveillance outputs. The supporting data and SSS follow: they are not the initial drivers for the surveillance systems. For example, during the London 2012 Olympic and Paralympic Games, the development of an emergency department SSS was given a higher priority because of the need to establish if visitors to the Games (particularly international visitors who may not consult a general practitioner) became ill and required emergency care [21]. In a similar need-driven way, the emergency department surveillance system in France followed the necessity (identified in the 2003 heatwave) to monitor, in real-time, the extreme effects of heat [22]. Therefore, while in both these instances, the individual events gave impetus to (and resources for) the development of the systems (with both continuing to provide surveillance for a wide range of public health threats), a general public health need can also be a driver for developing such systems.

We advocate that real-time SSS should be designed and operated to serve multiple public health objectives and not established for a single disease/discrete mass gathering, as once established the inherent nature of syndrome-based reporting has wider utility. An increase in ‘difficulty breathing’ in a population may indicate an infection, but may also indicate an air pollution event – thus the surveillance team need to be linked with a variety of health protection practitioners to provide intelligence widely.

Often forgotten is the need for reassurance about lack of impact on the public's health of an event or mass gathering. Such reassurance is frequently required throughout an event and in real-time for highly publicised events: real-time syndromic surveillance can provide information that forms a key component of this reassurance. This is valued by those managing the public health response to the events [14].

### Lessons: importance of relationships

For sustainable and successful SSS, we argue that relationships are of crucial importance, both with the data providers and with the public health users of surveillance outputs. Yet their importance is rarely described in the literature.

Similar to some traditional surveillance systems such as laboratory surveillance, the relationship between SSS and data providers is crucial to a well-functioning system, and understanding the nature of this relationship is key. For example, in England the role of the National Health Service (NHS) 111 telehealth triage service is to safely manage and triage callers who need advice about subsequent care, not to perform public health surveillance (i.e. provide data for SSS). Often anonymised data are transferred by such data providers to the syndromic surveillance team for the ‘public good’ and at no cost to the syndromic surveillance team. Thus, trust between the data provider and the syndromic surveillance team is important, and needs to be proactively worked upon/strengthened (we undertake this by regular contact and joint steering meetings with the data providers, and ensuring that the data providers are fully aware of how the data are being used). The syndromic surveillance activity needs to be mutually beneficial for the data provider and the syndromic surveillance team; the data providers need to be assured about data security and that the syndromic surveillance team will not compromise the data providers by releasing interpretation on/raw data without the providers being aware of this. It is possible that one breach of trust/release of data undermining the data providers could disable a SSS. We feel this issue of relationships and trust between teams is seldom mentioned in the field of syndromic surveillance but is fundamental to underpinning sustainable systems.

The links between SSS and public health users of such data are also critical and outputs from a syndromic system should rarely stand alone. In order to provide interpretation of outputs, the syndromic team need to have good ongoing relationships with disease experts, or those public health colleagues leading an incident. It is important to strike the right balance in the interpretation of SSS outputs, reflecting the strengths of the immediacy of the signals generated but also the limitations with regards to aetiological specificity. Thus, e.g. syndromic surveillance outputs should not purport to provide unilateral interpretation on the impact of a flooding incident (for which other intelligence is available such as microbiological surveillance reports and situational reports of local outbreaks), but rather should be included as a part of an integrated output by those leading the incident response. However, an incident director still needs an output from the syndromic surveillance service which is interpreted by epidemiologists with syndromic surveillance knowledge and working within a public health context. A line list of multiple statistical alarms for multiple syndromes will be of little help to a busy incident director, and in the planning for the 2012 Games, this was emphasised by those leading the public health response and was addressed by the introduction of a syndromic surveillance risk assessment system [7, 14].

### Lessons: surveillance system ‘infrastructure’

SSS inevitably vary in the resources that are required/available to run the systems. We have previously stressed that the focus should be on the public health utility. In order for this to happen, there should be input from conception of the SSS from those with clinical and public health/epidemiological and statistical expertise in addition to those with informatics and information technology expertise. We feel that the value of the integrated whole is greater than the sum of the parts for those systems encompassing these complementary disciplines.

The provision of health care services across the world differs. In England, health care provision is via the NHS and is free at the point of delivery. This is fortuitous for syndromic surveillance in that we are able to work with health service managers to obtain national real-time data (e.g. NHS 111 data encompass the whole of England). This is additionally helpful for surveillance as we only need to negotiate with one organisation, even if other stakeholders need to be consulted, and is a public service that has public health responsibilities in addition to clinical ones (in contrast with countries with numerous private providers of clinical services).

From experience, once the data governance requirements (including data sharing agreements) are in place to support ongoing access to syndromic datasets, it is key that nothing additional is requested from health care data providers; this assures sustainable and resilient systems. For example, we do not ask health care staff to code differently, use additional codes or to provide additional details about risk factors. Feedback from data providers suggests that any additional burden placed on front-line healthcare providers will result in an unsustainable system that will not deliver data. To prevent this, we ensure that the extraction and transfer of surveillance data is automated and occurs in the background of the service provider system, thus not interfering with, or adding additional time to the health care consultation.

One size, however, does not fit all. SSS in individual countries will depend upon their health care infrastructure, and long-term systems need to build upon the data resources available. For example, countries with city-wide health care organisations often have city-wide surveillance systems; and in developing countries/those countries with changing health care infrastructure, social media-type syndromic surveillance (which is not dependent on health care infrastructure) may have particular value.

With ever-increasing data available, there is the tendency to focus on more and more data analyses and on algorithms as part of the SSS infrastructure. We suggest the focus should primarily be on the questions and use for real-time syndromic surveillance, and the acquisition of larger and more diverse datasets and algorithms should follow to support, rather than the surveillance being driven by the data sources.

## Questions and issues still to be tackled in syndromic surveillance

We have divided these questions/issues into those relating to the purpose and aims of, relationships with colleagues and infrastructure of SSS.

### Issues still to be tackled: purpose of SSS

Much of the available syndromic surveillance literature is about public health incidents that have been detected by SSS or a description about the health impact of incidents. There is little literature about the types or characteristics of incidents/outbreaks which *cannot* be detected by SSS and the detection limitations. We need to be clearer with public health colleagues about the detection capabilities and limits of existing SSS.

To do this, we need to develop suitable methods for assessing the sensitivity and specificity of SSS. For example, when describing the performance of a single laboratory test for a single organism, it is relatively easy to calculate and communicate a measure of sensitivity and specificity. In comparison, for a SSS measuring multiple syndromic indicators (ranging from vomiting to conjunctivitis) for a variety of diseases of public health importance, this becomes much more complex. There needs to be a methodology for the calculation of sensitivity, specificity and positive predictive value which could be used across different types of SSS in a variety of countries and for different syndromes and diseases.

We feel there should be an increased clarity with public health users of information from syndromic systems about the types of condition which are amenable for monitoring/detection via syndromic surveillance. One important aspect to consider is the specificity and sensitivity of the clinical syndrome being reported for the specific disease that one may wish to draw conclusions for. For example, monitoring ‘hay fever’ (and codes which may be indicative, e.g. allergic rhinitis) gives a useful indication of seasonal trends in allergic rhinitis. Many conditions may present, or be coded initially, in a more non-specific way (e.g. people with Legionnaires’ disease could present with, and thus be coded by clinicians as cough/chest pain or difficulty breathing or headache or fever or a combination; conversely Legionnaires’ disease is likely to be a minor component of people presenting with ‘cough’ or ‘difficulty breathing’). Most syndromic systems will not be able to detect an early increase in, or reliably identify trends in such diversely presenting diseases or attribute any observed increase to a specific disease – and laboratory confirmation remains the mainstay of surveillance for such diseases. Additionally, SSS were originally developed for the purpose of detecting bioterrorist and deliberate release events. While this purpose remains important, there needs to be clear understanding of the utility of these systems in detecting such incidents. Very few SSS are likely to be able to detect small numbers of early cases arising from exposure to a biological or chemical agent, particularly when they present diversely in terms of their clinical and epidemiological characteristics.

Most SSS demonstrate what is happening now or over the previous days. The incident cases are frequently compared with those expected for the time of year to understand seasonal trends, increases in activity, etc. This information is of help for those running health care systems, but what may be of increased value is forecasting the potential impact of short-term changes in the disease burden or clinical activity on other parts of the health care system in the near future, e.g. using telehealth cough calls to predict subsequent increases in hospitalisations for pneumonia. Work exploring practical forecasting using conditions monitored by SSS is still in its infancy. Yet if SSS are shown to be of value in forecasting, the real-time nature of SSS could make such short-term predictions of great value for those managing surges in, and seasonal pressures across, health care services, e.g. in preparing the health and care services for upsurges of influenza-like illness or viral gastroenteritis.

### Issues still to be tackled: importance of relationships

The English SSS are based upon data which are not mandated, and under clear governance arrangements (between those running the syndromic systems and those providing the health care usage data) that the data are to be used for public health purposes only. Those running SSS are often asked for the ‘raw data’ by a variety of organisations, e.g. academia, health care providers, media, etc. There is thus a balance between the drive for data openness and sharing, with data providers who may not be willing to provide the data for SSS if they then have no control over the subsequent use and interpretation, and their data are simply ‘posted’ by those running SSS. This balance and agreement about subsequent data sharing, if handled incorrectly, could undermine systems, and thus needs to be clearly agreed and constantly reviewed.

### Issues still to be tackled: surveillance system ‘infrastructure’

Many countries have SSS, and in some, these systems are multi-setting (encompassing, e.g. GP attendances, emergency department attendances and telehealth calls). What is unclear is how many SSS is ‘enough’ in terms of data feeds for a comprehensive, optimally performing and cost-effective syndromic surveillance service. As more potential data streams become available, there needs to be clarity about added value of new potential sources, e.g. social media and Internet search data, and indeed whether some syndromic data sources could be dis-established because they add little to the public health intelligence. There is also little consensus on how to combine multiple data streams to get the maximum intelligence from a number of different data sources (e.g. ambulance dispatch data and out of hours general practice consultations) and across multiple syndromes (e.g. can cough in one system be usefully combined with fever from another).

The commonly used definition of syndromic surveillance is quite long; however, it does include a good description of the purpose and potential data sources [23]. There needs to be some consistency and increased rigor in the literature about the use of the term. The term syndromic surveillance has been used to describe work to explore the statistical relationship between presenting symptoms (often based on modelled data) and theoretical *potential* detection capabilities, which have not been acted upon (because the work is theoretical). We contend that there should be a clearer distinction between this type of modelled theoretical work and SSS working in practice. Such SSS use ongoing, often passive data collection, but actively support public health actions as part of a wider public health service.

## Summary

Real-time syndromic surveillance is not new and now forms a component of the surveillance for communicable and environmental diseases, and for situations that have the potential to impact adversely on the public's health, in many countries. SSS is however still in its infancy compared with traditional surveillance methods. It is still evolving and we have much to learn about where SSS adds value and where it does not. Most literature in this area is about data, algorithms and potential detection capabilities (often using synthetic data). We now need to move on to defining the public health needs for SSS and designing systems to best meet those needs. We feel that SSS is a good example of the use of large datasets, but contend that the focus for sustainable and useful systems should primarily be on the added value of such systems and the importance of people working together to maximise the value for the public health of syndromic surveillance services.
